# Loom: A Modular Open-Source Approach to Rapidly Produce Sensor, Actuator, Datalogger Systems

**DOI:** 10.3390/s24113466

**Published:** 2024-05-28

**Authors:** William Richards, John Selker, Chet Udell

**Affiliations:** OPEnS Lab, Oregon State University, Corvallis, OR 97330, USA; richawil@oregonstate.edu (W.R.); john.selker@oregonstate.edu (J.S.)

**Keywords:** environmental sensing, in situ sensors, Loom, low power, low cost, Arduino, datalogging, internet connectivity, Wi-Fi, 4G LTE, radio telemetry, power conservation, STEM education, water monitoring, soil monitoring, atmospheric monitoring, agricultural monitoring, scientific monitoring, open-source, real-time data, off-the-shelf sensors, microprocessors, Maker community, customizable sensors, OPEnS, Oregon State University

## Abstract

In the face of rising population, erratic climate, resource depletion, and increased exposure to natural hazards, environmental monitoring is increasingly important. Satellite data form most of our observations of Earth. On-the-ground observations based on in situ sensor systems are crucial for these remote measurements to be dependable. Providing open-source options to rapidly prototype environmental datalogging systems allows quick advancement of research and monitoring programs. This paper introduces Loom, a development environment for low-power Arduino-programmable microcontrollers. Loom accommodates a range of integrated components including sensors, various datalogging formats, internet connectivity (including Wi-Fi and 4G Long Term Evolution (LTE)), radio telemetry, timing mechanisms, debugging information, and power conservation functions. Additionally, Loom includes unique applications for science, technology, engineering, and mathematics (STEM) education. By establishing modular, reconfigurable, and extensible functionality across components, Loom reduces development time for prototyping new systems. Bug fixes and optimizations achieved in one project benefit all projects that use Loom, enhancing efficiency. Although not a one-size-fits-all solution, this approach has empowered a small group of developers to support larger multidisciplinary teams designing diverse environmental sensing applications for water, soil, atmosphere, agriculture, environmental hazards, scientific monitoring, and education. This paper not only outlines the system design but also discusses alternative approaches explored and key decision points in Loom’s development.

## 1. Introduction

Research is shaped by what can be observed, and thus instrumentation. The need for and lack of robust, automated, in situ environmental sensing methods across the spectrum of geophysical science is well documented [1,2,3]. On-site automated devices remove the need for on-site data collection by hand and provide critical ground-truth data that can significantly complement and correct remote sensing approaches. The documentation in pursuit of these environmental monitoring systems across several applications including agricultural, atmospheric, hazards, and health are numerous [4,5,6,7,8]. A common theme that unifies all these approaches is starting from scratch—integrating specific components ad hoc to yield an application-specific solution. When designs are ad hoc, the time and effort required to directly translate innovations from old designs into new ones are significant. Innovations and discoveries can be too easily lost in literature. Additionally, an increasing body of research promotes engaging multiple sources of knowledge such as formal science, local communities, and Indigenous knowledge in participatory co-design, management, and maintenance of environmental monitoring technology [9,10,11,12,13]. We propose an open-source, general-purpose, modular, application-inclusive approach that not only produces data for environmental research, but applications that facilitate education and co-design of sensor systems.

Our versatile software library and hardware management system, called Loom (https://doi.org/10.5281/zenodo.11318101, accessed 23 May 2024), sets itself apart from many commercial dataloggers in four major ways. By using off-the-shelf hobbyist electronics, the overall cost required to build and deploy dataloggers is reduced. The modular nature of Loom also provides many opportunities for customization and reconfiguration. Including support for actuators enables devices to mechanically respond to the environment, like turning on a sprinkler. Finally, support for Max8 from Cycling’74, a graphical real-time programming environment, enables interactive sensor activities for education and training.

Loom is a framework designed to significantly decrease the amount of time and effort to prototype new environmental monitoring systems, collect data, and interact with sensor-responsive controls. The systems we focus on all utilize some subset of the following typical components: sensors, actuators, radio communication (telemetry), media for data storage, internet connectivity, timing mechanisms, power management, and debugging. All this functionality is combined and deployed on a small low-memory low-power microcontroller.

Most individual hardware components that are integrated into Loom have example code and application notes from the manufacturer. The manufacturer notes and documentation are written by engineers for engineers. It takes training and experience for users to navigate and apply this documentation effectively. A typical approach is to start with these individual resources and integrate components one by one, testing for functionality with each step, until the complete prototype is produced.

One key aspect reducing the time between concept to completion of new prototypes is the extensive programming effort required to integrate each component yourself. This can also pose a barrier to those possessing other relevant skills like electronics, mechanical engineering, and environmental science to modify or produce their own systems who require specialized programming assistance. Instead, what if every component for each project was modularized into a reconfigurable framework? One that required only minimal programming experience to use. Only the novel components unique to new projects would need to be developed from scratch and modularized. Every subsequent project becomes potentially faster to prototype as more options become available to draw from.

There are increasing numbers of both commercial and open-source environmental monitoring solutions. Many systems such as EnviroDIY/ModularSensors [14], Open Source Building Science Sensors (OSBSS) [15], and the CR-family of devices from Campbell Scientific [16] can provide real-time online data access to anyone around the world with an internet connection. Real-time data are important for several reasons. They enable users to stay up to date on the status of the environment, provide feedback and warning signals for major events, automate internet-connected services, and help technicians assess maintenance and functionality of the sensing systems without traveling to remote field sites to inspect by hand. These datalogging systems also have the potential to significantly increase the accessibility of important monitoring and educational tools for historically underserved and marginalized communities alike, leading to more robust and sustainable citizen science efforts, representation of traditional ecological knowledge in academic research, and broader exposure of science practices and instrumentation to more diverse future-career scientists [17].

The benefits of Loom over other open-source solutions arise when examining how sensors are commonly integrated with commercial dataloggers. For example, the CR1000x Campbell Scientific datalogger supports various common protocols (see list in reference; however, the implementation for a given sensor is left up to the user to create themselves. The datalogger requires that the complete sampling loop be written in Campble Scientific’s proprietary language, CRBasic [18]. This approach, while versatile, is restrictive, and significant initial effort is required to simply read data from a given sensor. Loom, on the other hand, utilizes Arduino, a self-described “easy-to-use software and hardware package” [19]; the predefined implementation for each sensor within the framework allows users to upload their code and expect it to work as-is. Additionally, the open-source nature of the system allows anyone to integrate a new sensor at any point to improve the overall user experience for everyone.

Loom was started in 2016 shortly after the establishment of the Openly Published Environmental Sensing Lab (OPEnS) at Oregon State University. The mission of OPEnS is to provide tools and engineering expertise to researchers in environmental science to overcome limitations in research through new open-source instrumentation. A cursory survey of open-source environmental sensor design yielded three major approaches: (A) starting from scratch as described above; (B) adopting existing open-source platforms like EnviroDIY, which are tailored towards specific applications; or (C) commercial systems like Campbell Scientific, which were not open-source or extensible by design. Other open sensor platforms like Seeed Studio [20] and DFRobot [21] were not as optimized for our low-power environmental sensing requirements at the time.

The OPEnS lab would eventually need to serve over a dozen monitoring initiatives across a variety of applications such as agriculture, water quality, hazard detection, and facilitating educational sensor workshops for professionals and students. Furthermore, funding for student design staff was limited. Loom is structured for a small team of programmers who support constellations of larger multidisciplinary teams focused on tackling unique electrical and mechanical challenges specific to each project. This has proven to be more efficient than hiring a team of programmers for each project working independently from each other. While our approach may seem typical from the perspective of industry, this model was not as obvious for our small university lab needing to significantly scale from a few ad hoc projects. A range of low-power, low-cost, modular integrations of both off-the-shelf and custom peripherals, including sensors, actuators, datalogging formats, internet connectivity (including Wi-Fi and 4G LTE), radio telemetry, timing, debugging, and power conservation hardware, have been developed over six years across fourteen different environmental sensor applications. This approach has proven effective for our needs and sharing these methods could be beneficial for the broader community.

The main objectives of this paper are as follows:

- Detail our methods and highlight strengths and weaknesses of this approach.
- Provide context for why a modular approach to software and hardware facilitates rapid prototyping of new systems.
- Demonstrate the versatility of Loom to accommodate various contrasting applications without substantial modification to the system itself.
- Demonstrate usability of Loom through examples of systems created by undergraduate design students.
- Highlight unique aspects of this system compared to existing solutions (e.g., actuator control, STEM education applications, user-friendly error logging, modularity).
- Present some novel applications that engage students and the public with environmental data activities for education and co-design.

For convenience, a list of non-standard words in addition to all acronyms and their expanded form are provided in Table A6.

## 2. Materials and Methods

**Figure 1:**
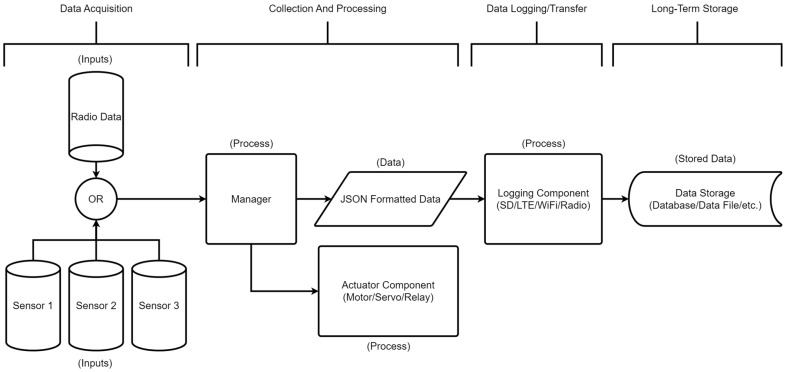
High-level overview of the data path Loom utilizes to collect, transmit, and store data. Each component of Loom can be categorized into one of the 4 stages above.

The Loom framework is modular, grouping functionally similar building blocks together. Each of the functional groups can be represented as a stage in a data pipeline in Figure 1. The first stage is the Data Acquisition stage. In this stage, the device has two configurations. The device can be configured to read data from various sensors attached to the device and pass these data to the next stage. Or it can be configured as a hub in which case it will relay sensor data transmitted from other devices via radio and pass along to the next stage. The next stage, Collection and Processing, formats raw sensor data into a JavaScript Object Notation (JSON) [22] document. If actuators are present, this is also the stage at which they would be set to new physical states. Then, the Data Logging/Transfer stage either stores data locally or transmits data over the internet/radio to a long-term storage location. Finally, the Long-Term Storage (output) stage represents the collected data logged to a database, file, or other non-volatile storage where further data analysis can begin. Since Loom follows this generic workflow, it is applicable to many different projects. As such, Loom undergoes extensive and diverse reliability testing. When bugs arise, we can quickly find and resolve them for all projects at once. Each of the sections below highlights a specific building block of Loom. They all work together to create the overall structure that provides Loom with its flexibility.

## 3. Hardware

**Figure 2:**
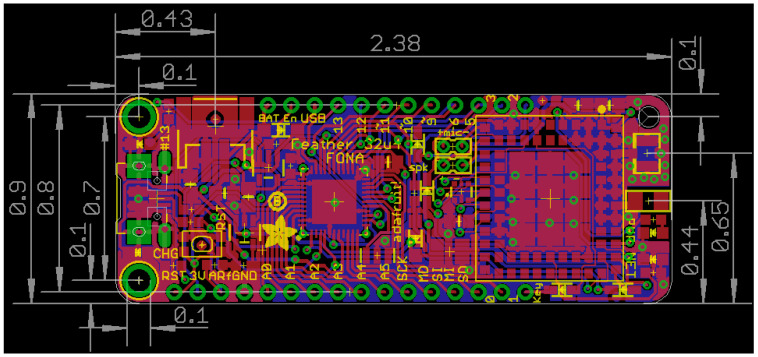
This shows the standard formfactor for all Feather M0 type boards [29], showing that all different versions of the M0 will always be interchangeable.

The hardware of the Loom architecture is built on the Adafruit Feather M0 family [23]. The Feather M0 is equipped with the SAMD21 microprocessor [24]. SAMD is the manufacturer’s design name for their general-purpose microcontroller. This low-cost microcontroller collection has several key advantages that make it well suited to our application. The Feather family comprises a variety of peripherals paired with a microprocessor, each providing functionality, including Long-Range, Low-Power Wide Area Network (LoRa) Radio [25], Wi-Fi [26], Bluetooth [27], and SD logging in the form of the Adalogger [28]. These configurations are provided on a formfactor called “Feather”, which has a dimension of 0.9″ × 2.0″ with 0.1″ holes at each corner (shown in Figure 2), a 16-pin header row spaced 0.8 inches away from a 12-pin header row, and a consistent pinout configuration enabling easier swapping across different boards adhering to the same layout [29]. This specification has increased in popularity, even being adopted by other high-profile companies in the community including Particle.io and SparkFun (Boulder, CO, USA). The significant advantage is that any product adhering to this formfactor and pin connection configuration reduces the development overhead to integrate with the Loom system and vice versa.

The choice of microprocessor useful across environmental monitoring projects necessitated prioritization of energy conservation, as many sites are remote, and may not have sunlight (e.g., forests), so the ability to run for a year on small batteries was a key design criterion. While there are other popular and more powerful microprocessors on the Feather formfactor like the SAMD51 (Microchip, Chandler, AZ, USA), the processor used the Feather M4 series of boards by Adafruit (New York, NY, USA) and the ESP32 developed by Espressif Systems (Shanghai, China). We determined the SAMD21 provided a good balance between processing power, compatibility with peripherals, and energy consumption. The SAMD21 32 kB of random-access memory (RAM) and 256 kB of flash increased utility over other popular Arduino-programmable chipsets including the ATmega32u4 (Microchip, Chandler, AZ, USA). The longstanding TI MSP430 (Texas Instruments, Dallas, TX, USA) was also considered for its attractive balance between ultra-low power consumption, high-speed processing capabilities, and industry reputation. It was excluded from consideration primarily due to the lack of compatibility with the Arduino Integrated Development Environment (IDE).

The Arduino IDE is prominent in the target Maker, do-it-yourself, hobbyist community, and a preferred platform for programming and development. The synergy achieved by combining the SAMD21 (Microchip, Chandler, AZ, USA) microcontroller, Feather family component options, and our variety of custom Printed Circuit Boards (PCBs) has rendered an extremely adaptable platform for the purposes of long-term in situ datalogging across a variety of applications.

Producing our own microprocessor board was also considered. The cost to develop a custom board from scratch versus the advantages of having a company produce a general-purpose board, maintaining and sourcing parts over time, and taking care of the extra significant step of installing the Arduino bootloader required to program the microprocessor was compared. It was decided the limited lab staff time would be better spent on designing the project-specific aspects of the electrical and mechanical systems. Highly volatile supply chain issues that occurred during the COVID-19 pandemic validated this decision as we were able to still source many of these Feather M0 boards from suppliers, but it was impossible to source our own components for a time.

### 3.1. Sensors

The sensing component of Loom is represented in Figure 1 by the three generic sensors in the top left that are passing data into the Manager which is discussed further in Section 4.3. Many commercial and open-source environmental monitoring systems focus on a particular stratum of the geosphere (e.g., water quality, landslide monitoring, air quality, or soil moisture). Supporting projects across many strata requires a broad selection of sensors and supported protocols including analog devices, Inter-Integrated Circuit (I2C), Universal Asynchronous Receiver/Transmitter (UART), Serial Digital Interface at 1200 baud (SDI12), and Serial Peripheral Interface (SPI). The decisions as to which sensors to include in Loom have been determined directly by the research projects for which funding was received for development. This collection of sensors (as well as affordability, precision, resolution, and ruggedness) is a result of circumstances unique to this lab’s region, research faculty, expertise, grant budget, partnerships, facilities, and institutional strengths. While this collection will not serve as a one-size-fits-all solution, many applications are supported and are readily expandable to include other sensors. Table A1 in Appendix A contains a full list of supported sensors to date.

### 3.2. Actuators

**Figure 3:**
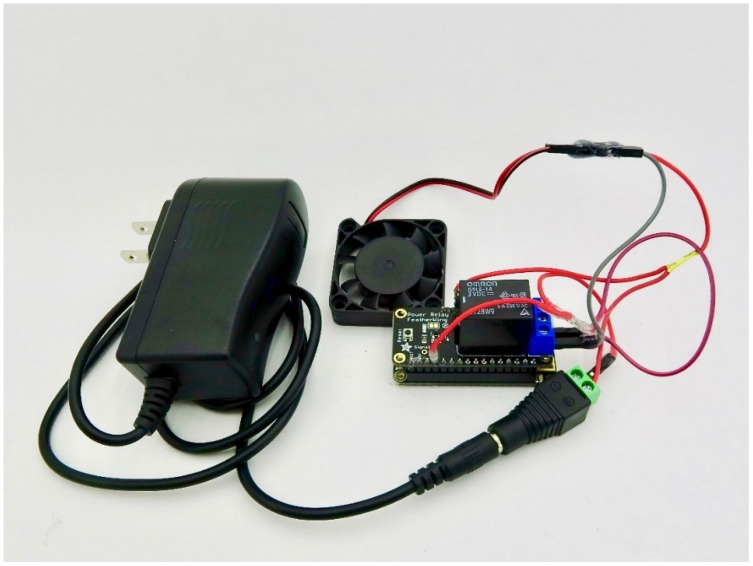
Shows a fan that can be turned on and off using a power relay shield. This is controlled using Loom on a Wi-Fi Feather M0 connected under the relay shield.

**Figure 4:**
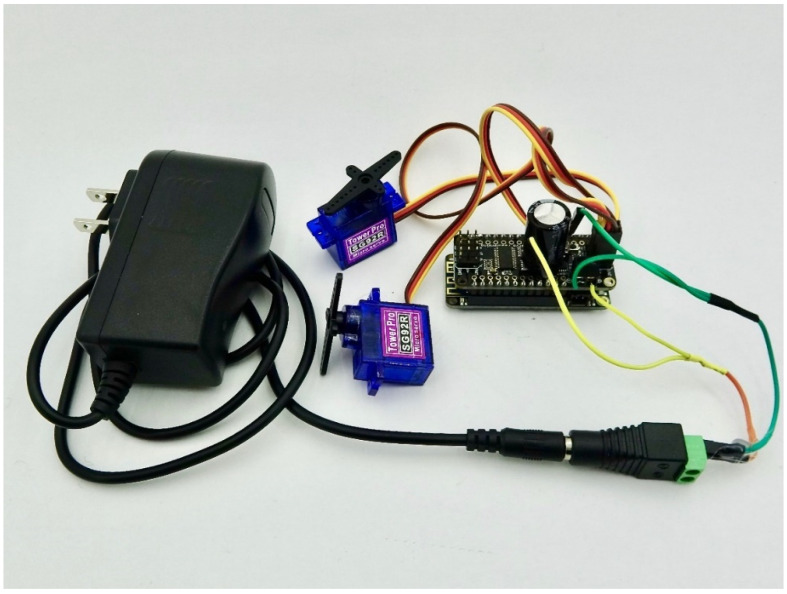
Shows two servos connected to a servo driver controlled using Loom on a Wi-Fi Feather M0 (connected under the servo shield).

**Figure 5:**
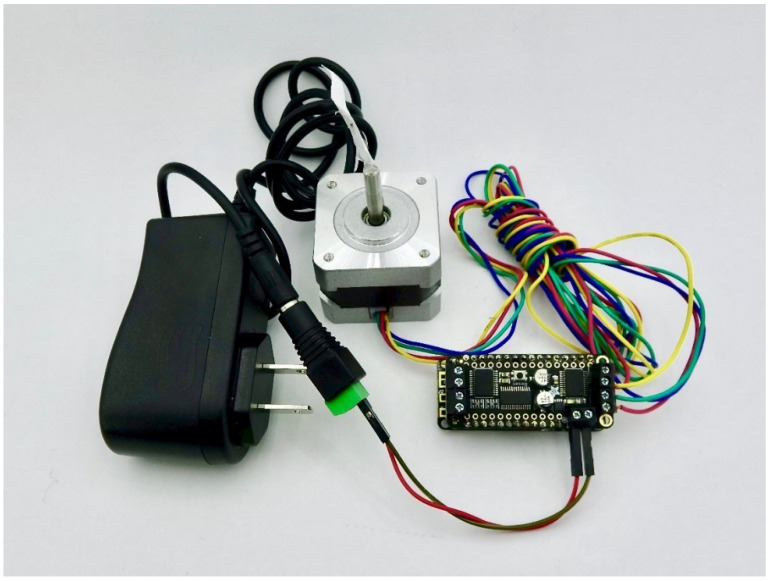
Shows a stepper motor being driven by a Loom-controlled motor driver via Wi-Fi Feather M0 (connected under the motor driver shield).

Actuators enable interaction with the physical world. Several common actuators are supported including relays, servos, and stepper motors (shown in Figure 3, Figure 4 and Figure 5). They are especially useful for when something mechanical needs to be performed in response to a sensor reading, like turning on irrigation when a soil sensor is dry or changing the position of a solar panel in response to sunlight. Actuator shields with Feather formfactors produced by Adafruit were chosen for ease of integration with the Feather M0 boards including latching and non-latching power relays [30]; 8-channel/16-channel Pulse Width Modulation (PWM) Servos [31]; and stepper and DC motors [32]. Each of the actuator types in Loom are generic, and as such, other hardware than listed here should also be compatible. Support for multicolored Light-Emitting Diodes (LEDs) called Neopixels [33] are also part of the actuator module and have been used as an informative visual status interface for some projects in the field.

### 3.3. Internet Interfaces

Loom supports three main forms of internet communication: Wi-Fi, Ethernet, and 4G LTE. Wi-Fi and Ethernet internet connectivity can be supported using off-the-shelf Adafruit products. The Wi-Fi Feather M0 or Ethernet FeatherWing’s [34] are plug-and-play and require no additional configuration on the Loom side. The SparkFun SARA-R4 [35] (U-blox, Zurich, Switzerland) board utilized by Loom to provide LTE internet connectivity requires some additional configuration steps to register a Subscriber Identity Module (SIM) card with a cellular network provider. 4G LTE hardware is significantly more expensive than Wi-Fi or Ethernet but allows for a broader distribution of internet-connected sensors if there is cellular data reception in the area. Each internet interface in Loom utilizes a NetworkComponent abstraction layer. This allows for all internet interfaces to be treated as the same type, increasing the modularity of the system. Some, all, or none of these interfaces may be used depending on the desired context.

It is significant to consider that in situ systems typically possess limited memory, processing power, bandwidth, and energy resources. Devices are typically battery powered. Various constraints challenge the feasibility of incorporating some network technologies like 4G LTE in all end devices, primarily due to energy consumption, cost, and scalability. Additionally, there is a lack of Ethernet, Wi-Fi, and 4G coverage in many field contexts. Data uplinks using Iridium satellite hardware like the SparkFun RockBLOCK Mk2 [36] for areas outside of 4G coverage have been accomplished for some projects [37], but cost has remained a barrier to feasibly implement as an option for Loom. These internet interfaces are one of several logging methods included in the data transfer stage in Figure 1. Telemetry using radios to transmit data from sensors in remote locations to a hub that hosts internet access is often used as a cost-effective approach for these situations.

### 3.4. Telemetry

Radio telemetry is often essential for in situ environmental monitoring [38]. It enables monitoring systems to relay data over distances (often via radio) to a hub hosting an internet connection. To address the challenges posed by low-power remotely deployed devices, we explored three different classes of radio hardware and protocols.

LoRa is designed for end-devices operating with limited battery capacity and transmitting only small data payloads. The SEMTECH SX127x [39] RFM 95 (Camarillo, CA, USA) radio chip was well positioned for our usage in Loom, especially because of the RFM 95 version of the Adafruit Feather M0 product. This option is a drop-in replacement for any preexisting system already utilizing a Feather formfactor. These radios are suited for small low-bandwidth payloads (max data rate of 300 kbps) to be transmitted between devices that are within a two-kilometer line-of-sight range.

Methods and circumstances to achieve two kilometers or even more range are numerous [40,41], but for application in hilly, wooded, and/or rainy regions, it is best to maintain conservative expectations of range. One such application of these radios is transmitting sensor data from an endpoint device that is out of Wi-Fi or 4G range to an internet-connected hub to relay data to the internet [42].

FreeWave (Boulder, CO, USA) radios [43], specifically the Z9 series, allow for a much longer range (exceeding 90 km) with a significantly higher data-transfer rate than LoRa affords, up to 4 mbps [44]. However, the price of these radios is high (around USD 500) [43]. Usage of these radios and integration with Loom has led us to determine these are a more stable and longer-range alternative to LoRa, but the cost makes them inaccessible for many open-source sensing projects.

Previous versions of Loom included the Nordic Semiconductor (Trondheim, Norway) NRF24L01+ [45] radio, which supports a higher 2.4 GHz bandwidth (250 kbps, 1 Mbps, and 2 Mbps), but a significantly shorter range (up to 800 m achieved under ideal conditions with a power amplifier). However, support for them has not yet been ported over into the newest version of Loom due to the priority of telemetry range over the data bandwidth [46]. Code from previous versions of Loom exists and reintegrating these radios is a viable option should the need for more bandwidth over shorter ranges arise.

### 3.5. Shields

In the Maker community, a shield refers to a plug-and-play PCB that serves as an extension to a host microprocessor board to interface with other peripherals. These PCB interfaces resemble shields and have been named thusly. They adhere to standardized formfactors of the microprocessor boards they adapt with (e.g., Feather), enabling Makers to extend their projects more easily with an array of functionalities like communication, motor control, display interfaces, amplifiers, sensors, and more—without requiring expertise in circuit design or soldering. The embrace of shields by enthusiasts and the open-source nature they embody have been catalysts for collaboration and further innovation, within the Maker community. In addition to commercial off-the-shelf shields, there are many custom shields designed by Makers to meet needs in the community that are not met by manufacturers. One significant custom Loom shield, Hypnos [47], was a result of identifying several core functionalities essential to environmental sensing and abstracting those into a single shield to drop into all projects.

### 3.6. Hypnos

**Figure 6:**
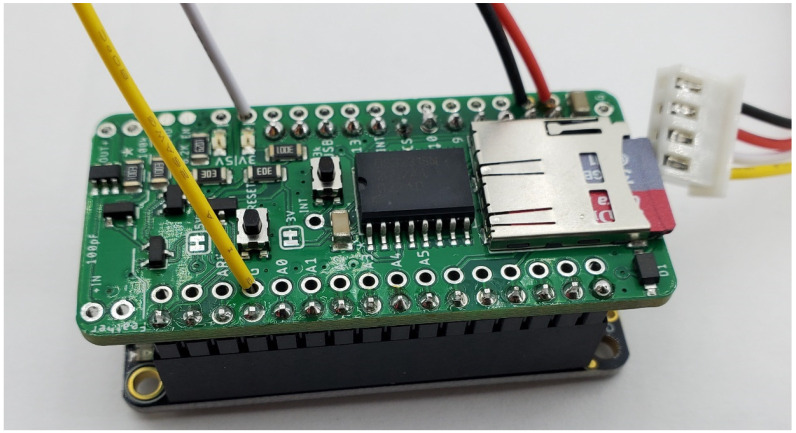
This figure shows the current version of the Hypnos board.

The Hypnos [47] is an SD-card logger, Real-Time Clock (RTC) and power-management solution in one easy-to-use shield (as seen in Figure 6). These three features are a necessity for most environmental sensing applications. Having a cost-effective solution such as Hypnos makes it extremely easy to mass-produce these shields for all the projects across our lab. A comprehensive explanation of the design and build instructions can be found in the cited HardwareX article.

### 3.7. I2C Multiplexer (TCA9548)

**Figure 7:**
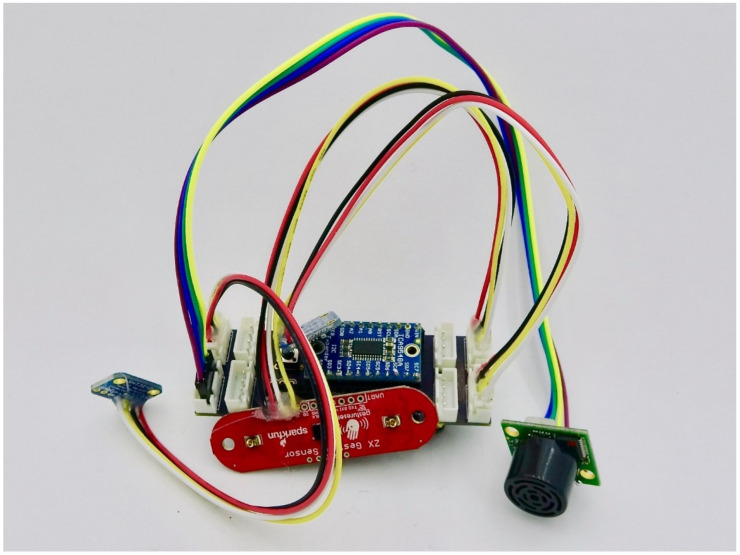
This figure shows the Loom custom shield for the TCA9548 with various I2C sensors including (left to right) SHT31 Temperature and Humidity, SparkFun ZX gesture (red), TSL2591 lux, Maxbotix sonar distance. See Table A1 for list of supported sensors.

Loom supports the TCA9548 (Texas Instruments, Dallas, TX, USA) multiplexer (breakout board shown in Figure 7). This allows up to eight of the same or different I2C sensors to be used at one time. It also enables automatic detection and “hot swapping” (e.g., dynamically swapping out components mid-operation) of sensors. When the Loom I2C multiplexer software component is used, each time a measure call is made, the list of possible I2C sensors will be queried and refreshed. If a new sensor is detected, it will be initialized at run time. This hot swapping feature has been useful for interactive workshop environments because many different sensors can be connected and experimented with on-the-fly without having to reprogram or restart the device or crashing the I2C bus due to not-acknowledgements from removed sensors.

### 3.8. Project-Specific Breakout Boards

**Figure 8:**
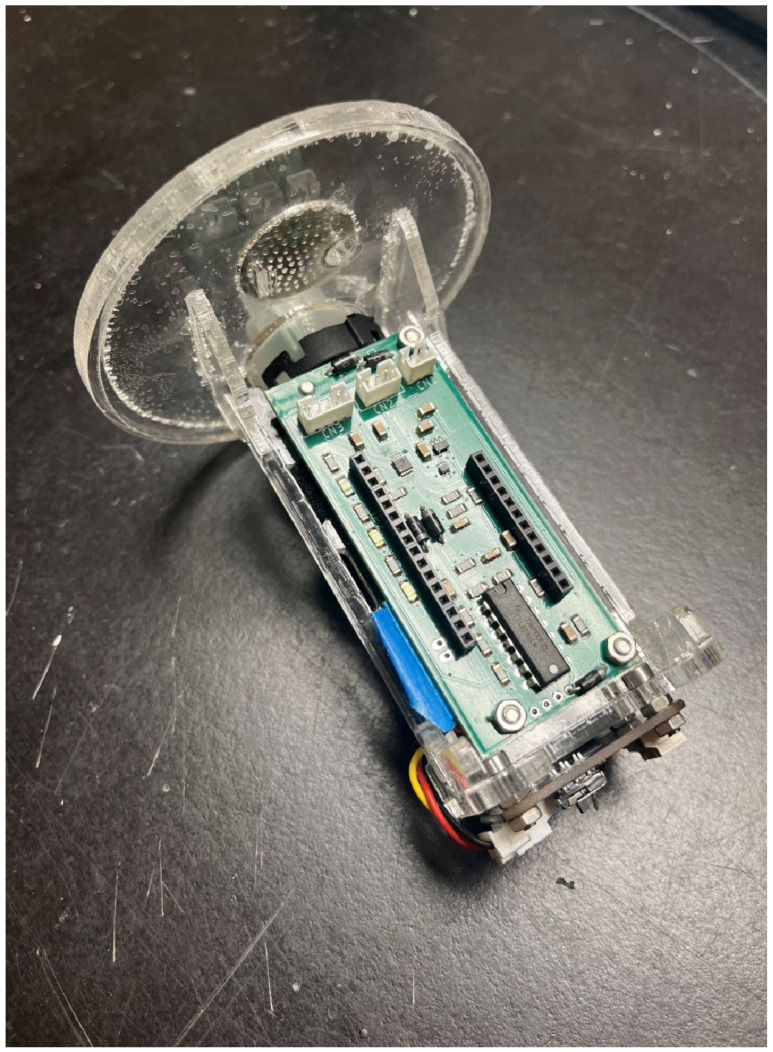
This shows the SmartRock expansion board.

For specific environmental sensor projects using Loom, the FeatherM0 and Hypnos boards are typically stacked together onto another custom PCB specifically designed for each project. This PCB routes the hardware connections Loom requires to interact with project-specific hardware peripherals. For example, the SmartRock [48] is a low-cost submersible sensor suite that monitors water depth, temperature, turbidity, and electrical conductivity of streams over time. Also known as a Sonde in the environmental and hydrological communities, these devices take the form of probes that are installed in water, which require a cable connection to a logging gauge on the surface. Alternatively, the SmartRock is designed for all components to be integrated underwater. This would mitigate common issues including vandalism of the gauge on the surface and connecting wires getting snagged or severed by branches and other floating debris. Figure 8 below illustrates the custom SmartRock PCB with project-specific components and connectors, including female headers adhering to the Feather formfactor convention to receive the Feather M0/Hypnos stack. More projects are described in the Section 5 below.

### 3.9. Course Shields

Specific configurations of the Loom system have been used in formal college courses and informal STEM education workshops. Course shields facilitate little-to-no assembly of the hardware for these students, enabling class activities to focus on key applied concepts more rapidly.

**Figure 9:**
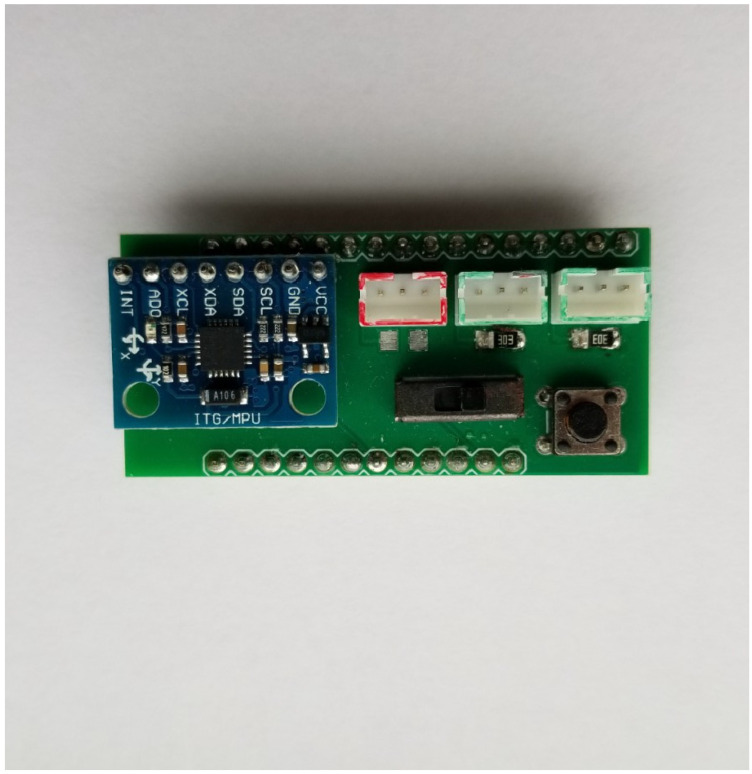
This shows the Wattson shield with MPU gyro and various other peripherals.

The Wattson (shown in Figure 9) connects a Feather M0 with Wi-Fi to an MPU6050 (InvenSense, San Jose, CA, USA) 3-axis accelerometer and 3-axis gyroscope for motion tracking, as well as three general purpose ports, a push button, and a switch. The general-purpose ports can be set as a combination of analog input, digital input, digital output, or Red, Green, and Blue (RGB) LED output. Pads for optional resistors are also on each port for adapting some sensors into a voltage divider circuit. The Wi-Fi interface provides wireless access to the real-time sensor data that can either be streamed to computer applications or uploaded to MongoDB cloud database. The shield has been used in an honors College Colloquium (Making Enchanted Objects), and a cross-listed College of Liberal Arts class (Sensor Technologies and the Arts).

**Figure 10:**
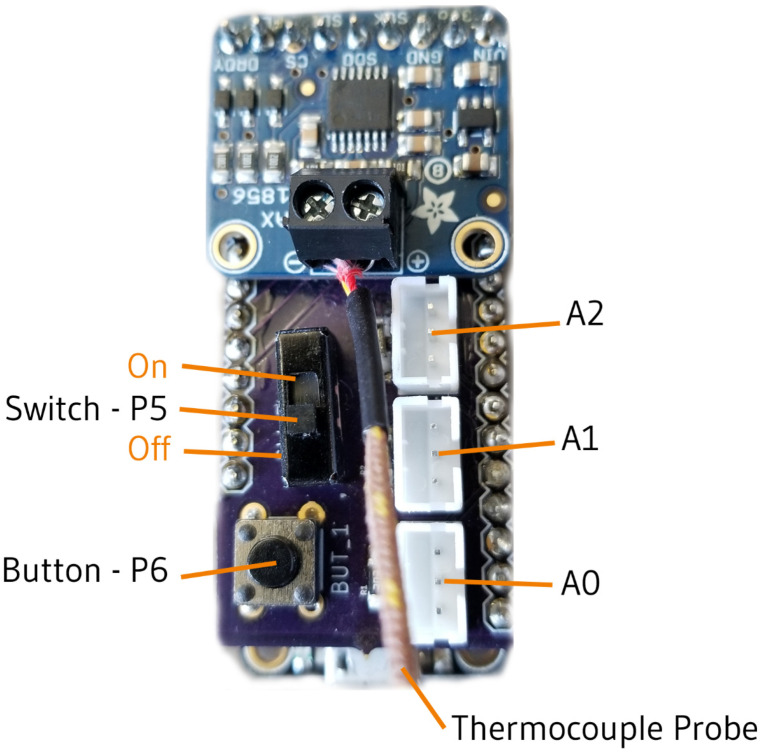
This figure shows the Loom-supported BEE222 shield which is used as a learning tool in courses at Oregon State University. The connectors labeled A0, A1, and A2 are connected to the pins on the microcontroller with the corresponding names. The same can be said for the switch and button labeled P5 and P6, respectively.

The BEE222 shield (as seen in Figure 10) is named after the introductory course for environmental sensing and adapts an Adalogger M0 containing an SD card data storage slot to three general purpose ports like the Wattson shield, 4-pin I2C sensor port, software serial port for a thermocouple amplifier, a push button, and a switch. This shield has been swapped onto an M0-WiFi Feather to enable online datalogging and connectivity. Plugging this M0-shield stack into a Feather Doubler enables students to connect a variety of other Feather-footprint devices such as a relay for turning devices on and off, motor controllers, and a DS3231 (Analog Devices, Wilmington, MA, USA) RTC to time samples and manage low-power sleep periods.

## 4. Software

Many open-source Arduino-compatible libraries that help simplify sensor prototyping exist. The closest in terms of functionality and scope is the ModularSensors library by EnviroDIY (Stroud Water Research Center, Avondale, PA, USA). Further comparative analysis of these systems will be discussed in Section 4. One key difference with Loom is the abstraction and modularization of similar workflows in environmental sensing systems. Loom is a modular framework, not a static algorithm. The user determines which components to include or leave out. They also determine when and how certain functions are performed. Even across a wide variety of applications, it is typical to follow a similar procedure in in situ environmental sensing.

Devices, when first powered on, will initialize all attached sensors, and run all first-time setup code. They will then enter the main software loop consisting of measuring data from sensors, taking the measured data and formatting it in a parsable format such as a comma-separated values (CSV) file or a JSON-formatted document. Sometimes an actuator will need to be set depending on the value of the sensors, like turning on a water sprinkler if a soil moisture sensor registers too low. They will log the formatted data to some medium like an SD card and/or publish remotely. We employ several protocols but the most used in Loom is the Message Queuing Telemetry Transport (MQTT) protocol. Finally, devices must power-off peripherals and enter a low-power sleep mode until triggered at some future time to reawaken and conduct the main cycle over again.

**Figure 11:**
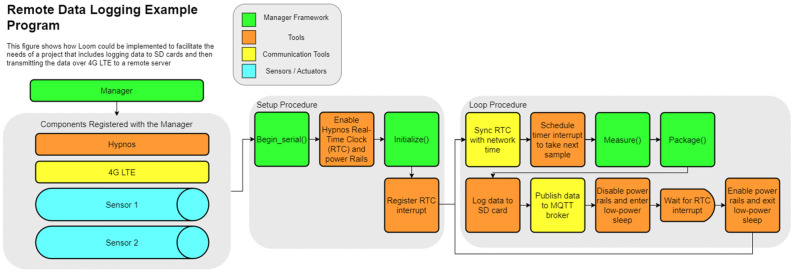
This diagram demonstrates how Loom can be used to create a system that collects data from sensors, logs data to the SD card, transmits the data over LTE, and then enters a low-power state until the RTC triggers an interrupt and restarts the loop procedure.

**Figure 12:**
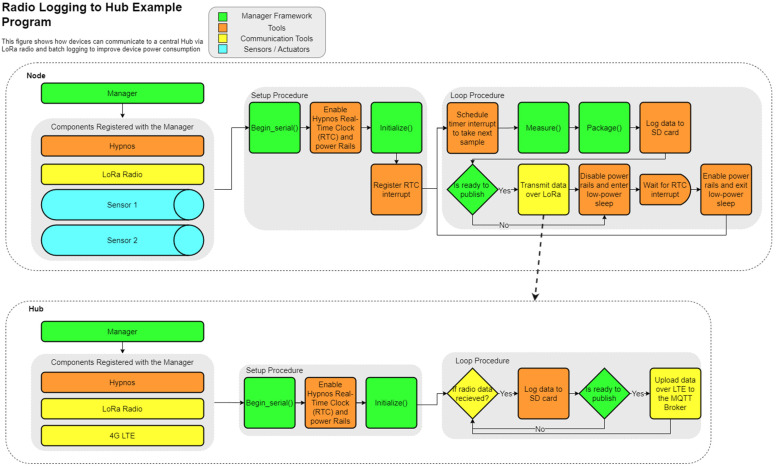
This diagram shows how Loom can be used to create a program where one device collects data and transmits data (like Figure 11) over LoRa radio to another device that then uploads the data to the internet.

The Loom framework standardizes this action chain regardless of what devices are being used within the system. The action chain need not use all the above steps. The chain can be conducted in different orders and can even be conditionally executed based on user specifications. Because of this organization, adding or removing components like sensors to an existing system becomes a matter of choosing whether to include a declaration of that component at the start of the code. Figure 11 and Figure 12 below show how different components of Loom can be integrated together to achieve different results while still maintaining a consistent structure. The specific components within the diagrams are discussed in the following sections.

### 4.1. Library and Board Profile

Many of the Loom modules depend on open-source libraries from the manufacturer or organization to function. Loom utilizes a modified Feather M0 board profile that automates installation of libraries and packages required to run Loom. This also allows modified versions of libraries to run alongside globally installed libraries so that patches can be created for libraries that do not inherently support the Feather M0 or do not behave entirely as desired. Having a local store of the libraries means that issues related to manufacturers pushing updates that could break our system can be avoided. This also allows us manufacturers to continue supporting and updating their products and for Loom to evaluate and accept these changes following due process.

### 4.2. Module Architecture

The architecture of each module inside Loom is derived from a generic module abstraction class. The following five methods are the main way one can interact with each module: initialize(), measure(), package(), power_up(), and power_down(). Which modules the user is going to need to utilize for each project cannot be predicted by the Loom developers. In a previous version of Loom [49], the developers chose to include every module and autodetect which modules were being used at runtime. This turned out to be an inefficient and infeasible approach given the microcontroller’s limited memory. Instead, Loom’s modules adhere to a universal blueprint, requiring new modules to implement the five above methods within this module abstraction class.

Consequently, all modules can be treated as one type, ensuring consistency, and enabling easy addition of new modules. This principle aligns with the concept of polymorphism in computer science [50]. Each module created within the Loom framework has a constructor for any parameters that may be required to facilitate different sensor configurations. These could include different I2C addresses, gain settings, communication pins, etc.

Each implemented module needs to have an instance of the Manager passed into it when it is created. This registers each module with the Manager. This allows the Manager to issue universal calls to those five methods, and all registered modules will execute those functions based on their own implementation (refer to Section 4.3). This allows for users to include any combination of modules without having to directly interact with the code of the individual modules themselves. This is the main footprint that allows us to create these action chains that we discussed in Section 2.

The initialize() method has the specific code that needs to run for each sensor to properly initialize and interface with the Feather M0. Each sensor unit will have its own initialization procedure and needs to be different for each module. For example, the ADS1115 analog-to-digital converter can have different gain settings that need to be set when the module is initialized to take accurate measurements. These kinds of functions are executed inside each module’s initialization code.

The measure() method is called when we want a specific sensor module to collect data from its associated sensor hardware. This function sends a request to each registered sensor to take a sample and store it for later use. This action is visualized in Figure 1 by the arrows connecting each sensor to the Manager process.

The package() method parses the collected data from each module into a JSON-formatted object and stores it within a global JSON document. This serves as a unified document that all modules can reference, pull data from, and reformat as needed. This stage is visualized in Figure 1 by the arrow connecting the Manager to the JSON-formatted data node.

The power_up() method must exist in all modules but is not necessarily used in all of them. This method is called as soon as the Loom device exits sleep mode. This function reinitializes any volatile settings and values that may have been lost when the device was powered off.

The power_down() method allows modules to safely shutdown before entering a low-power sleep state and cutting power to the sensor. This allows for better reliability in powering the sensors back up without issue. One such use case would include properly powering down and disconnecting I2C devices where pulling power could cause instability issues for the next cycle.

### 4.3. Manager

The Loom Manager maintains an abstraction layer that allows for seamless interaction with all peripherals and is key for coordinating system-wide operations. The Manager also has calls for initialize(), measure(), package(), power_up(), and power_down(). Instead of defining specific characteristics of each module, we are calling the corresponding function on each registered module and storing them in the Manager’s JSON document. The usage of initialize(), measure(), and package() can be seen in both Figure 11 and Figure 12 right before the data are logged to the SD card. The Manager also has a few unique functions.

When a new Loom module is constructed within our Arduino sketch, a register_module() method is called on the Manager and the instance of the module in question is passed in and added to a list. This list held within the Manager contains pairs of strings and modules. The strings store the module’s name while the module is a reference to the module with the given name. This act of registering the modules with the Manager allows us to be able to interact with all included modules by simply talking to the Manager. The action of registering each component can be seen in Figure 11 and Figure 12 within the “Components registered with the Manager” section.

The begin_serial() method tells the Manager to wait 20 s for the serial monitor to be opened before continuing. This allows the user to see the first several print statements that may be missed if the user takes time to open the serial monitor. The addition of the 20 s timeout in conjunction with the while(!Serial) call makes it so that no reprogramming is required when devices are deployed in the field. If the while(!Serial) command were present without the timeout and the device was powered on without a USB connection, it would hang in setup. This action occurs as soon as all modules have been registered as seen in the setup procedure section in Figure 11 and Figure 12.

The display_data() method prints the user-readable JSON-formatted data to the Arduino serial monitor or the supported Organic Light-Emitting Diode (OLED) display so that the user can see the collected data in real time.

### 4.4. Hypnos

The Hypnos software library ties all the hardware components of the Hypnos shield together (see [34] for details). Loom utilizes the SDFat [51] library to provide stable SPI SD card communication between the Feather and SD card. The files on the card are formatted using an incremental file system where each individual device cycle (device completely restarting) will log to its CSV file; all logging is facilitated by a single log call that formats and logs data to the SD card.

Loom also supports saving many sequential packets as a “batch” to be transmitted all at once to conserve energy that powering on internet components for each packet would otherwise use. This action can be seen in Figure 12 on both the *Hub* and the *Node* where the device waits until enough packets have been collected before transmitting. The Feather M0 has manufacturer support to enter a low-power standby state that will then wait for an interrupt on a given pin to wake back up. The Hypnos expands on this feature, utilizing the DS3231 real-time clock to schedule time-based interrupt signals to wake the device at a set sampling period. The next interrupt is scheduled at the start of each sample period (as shown in Figure 11), only after the RTC is updated with the current network time (if network components are attached). This is performed at the start to ensure consistent times between samples that are not affected by devices responding slower than normal. Hypnos are also able to programmatically sever power to all sensors when entering sleep, allowing the device to use even less power, and extending the battery life.

### 4.5. Integrating New Modules

For maintaining modularity and expandability, a standardized procedure for integrating new modules into the Loom framework called “Loomifying” was developed. Many manufacturers and developers provide example code and libraries to use their sensor on the Arduino platform. Loomifying starts with evaluating this example code or writing your own. Once the sensor and code are confirmed functional, additional checks and timeouts are added to this generic code as well as features to ensure system-wide stability over long-term usage and changing conditions (discussed in Section 4.10 below). This partial integration of example code and support functions with the other Loom code is assessed for long-term stability. Finally, the partial integration is formatted into its own Loom module structure referred to in Section 4.2. Once proven stable, the module is merged into the main build, making it available for use in any subsequent projects. Templates providing examples for different components like sensors, actuators, and internet interfaces are provided in the repository.

### 4.6. Actuation Integration

**Figure 13:**
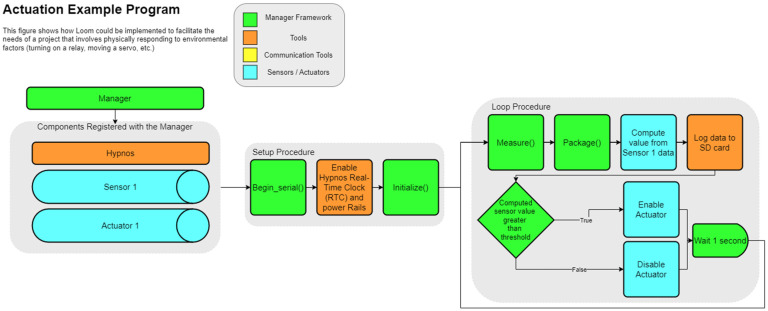
This diagram shows how Loom can be used to convert environmental stimuli into a physical response when a given calculated value is greater than or less than some threshold.

One of Loom’s many unique features that is often absent from other unified data-collection frameworks is the ability to control different physical actuators such as stepper motors, servos, relays, and LED Neopixels (see Table A3 for a complete list). These allow developers to easily invoke physical responses to the data they have collected or even allow them to be controlled remotely through other Loom-connected applications (Refer to Section 4.9 (Max)). An example application of the Loom framework to provoke such a reaction is illustrated in Figure 13. The integration of the actuators relies on a slightly modified module parent class aptly called the actuator. As a module itself, it has the same methods such as measure() and package() as any other sensor. However, it also has an additional control method. The control() method is overridden on each sub-actuator and provides the actuator specific functionality to control the connected hardware. Depending on the device, this could mean moving a servo or changing the color of some LEDS. A key-value pair sequence is provided to a given actuator’s control method to specify how we want to control the connected hardware.

### 4.7. Telemetry Integration

Radios in Loom are all derived from a basic radio abstraction class utilizing the RadioHead [52] library for reliable data communication. This class provides the footprint for functionality such as transmitting and receiving in addition to handling decoding of MessagePacks [53]. It is easier to integrate and use different radios if all radios are set up in a comparable manner. In this case, two other methods are specified for radio modules: transmit() and receive(). Loom also supports large packets over LoRa (a common issue using these radios) by splitting the packet payloads into sizes of less than 251 bytes (max LoRa payload size), making several transmissions, and reassembling the packet on the other side. This use case is again detected and managed automatically in Loom, further decreasing development time and issues writing code from scratch.

### 4.8. MQTT

**Figure 14:**
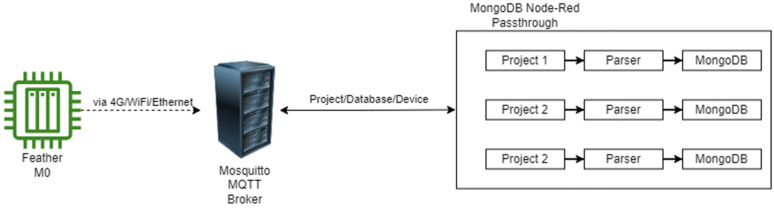
The process flow for data transfer from the Feather M0 device to MongoDB.

The MQTT protocol utilized within Loom allows for easy transmission and interpretation of data from many different devices and uses a publish/subscribe format. This restricts data to different topics that can be accessed individually. Loom’s application relies on using the open-source Mosquitto [54] broker to handle servicing MQTT requests. Each request is formatted where each component is separated by a slash; project name (if applicable), an arbitrarily defined database name, and finally, the device name concatenated with the instance number (e.g., Project1/WeatherChimes/Chime6). From here, we utilize a custom Node-Red [55] flow that is based on the root of the MQTT topic. The topic determines which MongoDB cluster to route the data. This transaction is illustrated in Figure 14. This allows organization of different projects across multiple clusters. These databases are hosted using MongoDB. While this may not be the best option for large datasets, it allows for easy data insertion and retrieval as all data are formatted as JSON before being pushed into the database. In addition, these MongoDB clusters offer convenient chart and data visualization tools. 

### 4.9. Max

**Figure 15:**
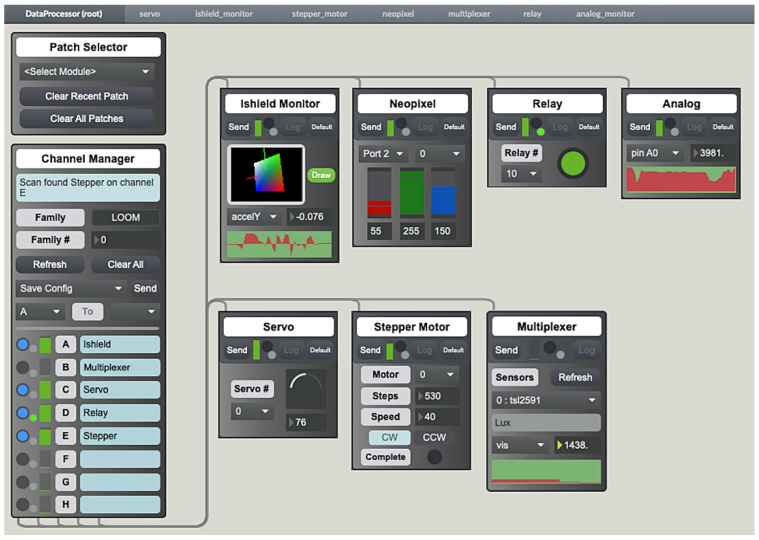
Max interface shows Wi-Fi connection client with real-time interactive sensor and actuator modules.

**Figure 16:**
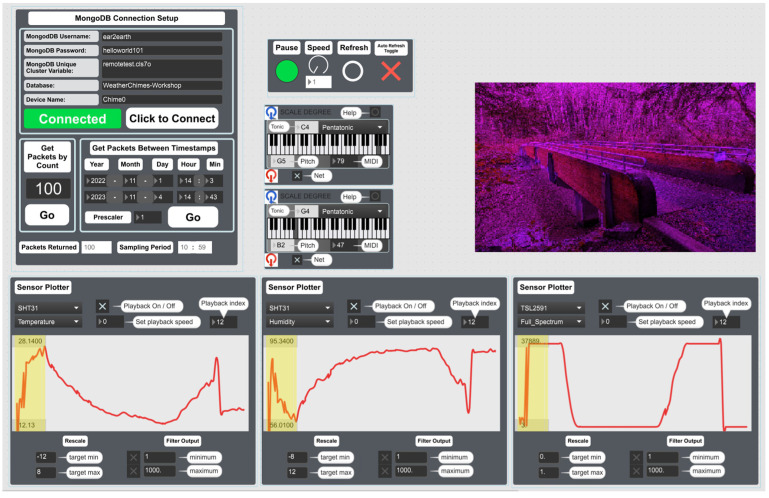
Max interface showing MongoDB database connection client, data plotter, data playback, sonification, and visualization module example. After connecting to the database, the user can parse individual data streams to plot over a defined time span and use those to shape audio signals and visual effects.

The application of Cycling’74 MaxMSP [56] also known as Max8 in the context of environmental sensing is one of the most unique features distinguishing Loom from other similar options. Max8 is a graphical programming environment that provides a more visual approach to viewing and interacting with real-time data in addition to controlling devices on a network as shown in the user interface (UI) in Figure 15. Loom devices have the capability to either connect to an existing Wi-Fi network or create an ad hoc local hotspot that can be connected to by a computer. Once connected, data visualization, interaction, control of different actuators such as servos or motors, and data sonification (translating data into audio signals) are accessible from any internet-connected Loom device. The application of such is shown in Figure 16. These devices may be connected on a local network, or from the field over 4G. This opens the possibilities for many different interactive educational activities used by students from middle school to college and scientists in workshops. The benefits of being able to see and manipulate tangible environmental data in real time makes an interactive approach to learning environmental sensors. One detailed account of using the Max applications for real-time environmental sensor and STEM education has been detailed in the WeatherChimes project section below. These applications have also been used significantly in two Honors College classes (Enchanted Objects, Electric Nature) and one cross-listed College of Liberal Arts class (Sensor Tech and the Arts) at Oregon State University.

### 4.10. Robustness, Troubleshooting Utilities, and Failing Gracefully

Having a robust protocol for communication and device control is crucial to running a successful datalogger. One main purpose of Loom is to prevent users having to completely reset the device to account for situations when sensors are disconnected mid-cycle, or when other unexpected edge cases occur. Instead of crashing the device, the system should be able to recover from errors and continue data collection. One typical issue is a system hanging from cases where I2C sensors fail to acknowledge during communication. Loom has support for I2C devices that disconnect mid-cycle and can determine whether it should no longer attempt to collect data from unresponsive sensors to prevent a hardware crash. Upon reconnection, the Loom Manager will attempt to reinitialize the sensor.

Another example arises with network communication not receiving an expected result. If Loom is unable to connect to a given network, the system should not wait indefinitely. Instead, it should retry a set number of times and then fail gracefully to allow us to continue collecting data, saving on other available media, and try to connect and publish again later.

**Figure 17:**
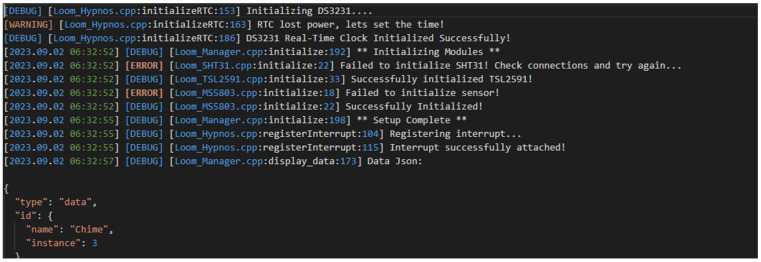
This figure shows the first several actions of a newly powered-on device utilizing Loom’s debugging framework to log the actions to a file. This mirrors what the serial monitor would print if connected to a computer and is logged to the SD card for evaluation during the entire lifecycle of the device.

In addition to preventive measures to ensure devices are running smoothly, a custom debugging framework to quickly diagnose and fix software was produced. This framework provides functionality for tracking and storing debugging information. The framework supports standard serial output as well as SD logging of all output. In addition, it is also capable of logging and tracking memory usage of individual functions inside of Loom to allow users to quickly find and fix memory leaks. Figure 17 illustrates an example log file produced by the logging framework.

In earlier versions of Loom, we utilized the SAMD21’s built-in watchdog timer (WDT) [24] (pp. 212–219) to handle fault recovery. The WDT will automatically reset the device if its timer has not been reset in a certain period, typically 8 or 16 s. However, this can also introduce several unpredictable issues once systems are faced with unforeseen field scenarios where the WDT has not been configured to respond to a presented edge case. Many instabilities were encountered specifically because of the WDT. Instead of relying on the WTD, Loom development focused on accounting for edge cases preemptively, and failing gracefully. If components stop responding mid-operation, the WDT is not relied on to reset the board.

## 5. Results

Due to modularity, Loom supports a variety of different projects and applications without significant redesigns to the system itself. Provided below are a few examples of devices produced by undergraduate engineering students utilizing Loom. These examples are not intended to provide an in-depth analysis of the data collected from each project. Each is explained and validated with data in high detail in their cited peer-reviewed articles and proceedings. A more thorough list of Loom-supported projects can be found in Table A5.

### 5.1. WeatherChimes

**Figure 18:**
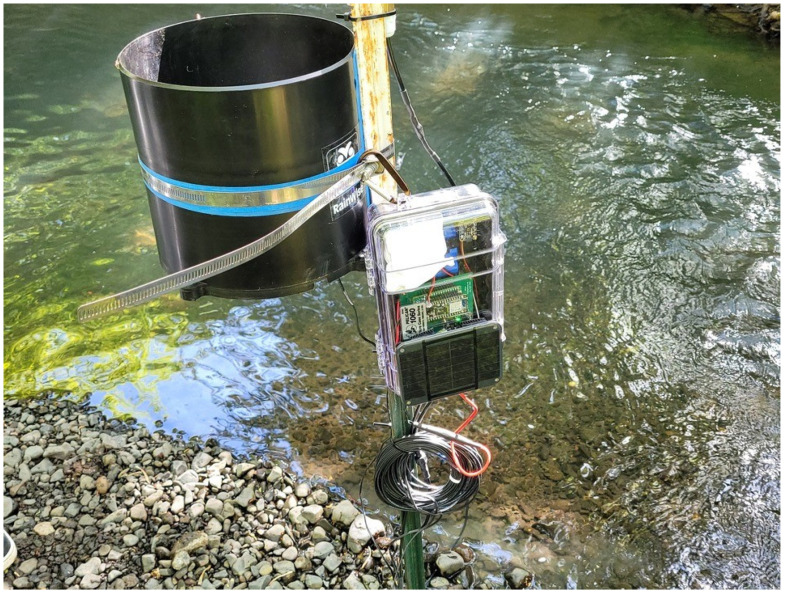
This figure shows a field-deployed WeatherChimes device.

WeatherChimes [57] (Figure 18) is a low-power in situ weather monitoring and sonification system, that enables near real-time access to environmental sensor data, including light, temperature, relative humidity, soil moisture, and rainfall anywhere with a Wi-Fi or 4G internet connection (Figure 11 shows a simplified version of this device’s firmware). It is being used by scientists, educators, and artists to obtain and interact with environmental phenomena in new and innovative ways. It is currently in use at Sitka High School’s Traditional Ecological Knowledge course, facilitating student observations of environmental impacts on Yellow Cedar. It is also in use in Hoonah, Alaska to measure water quality and rainfall at nearby reservoirs and to compare remediated versus not-remediated streams. Much of the local rainfall data are intended to supplement models tracking landslide risk in communities. WeatherChimes data and hardware have also been used in community and undergraduate STEM education and co-design programs. Some activities include transforming data into auditory signals, soundscapes, and visual art as part of educational workshops and college-level courses using the Max8 computer software (https://cycling74.com/products/max, accessed 23 May 2024) as shown in Figure 16. This effort yielded a peer-reviewed article with two undergraduate co-authors.

### 5.2. Dendrometer

**Figure 19:**
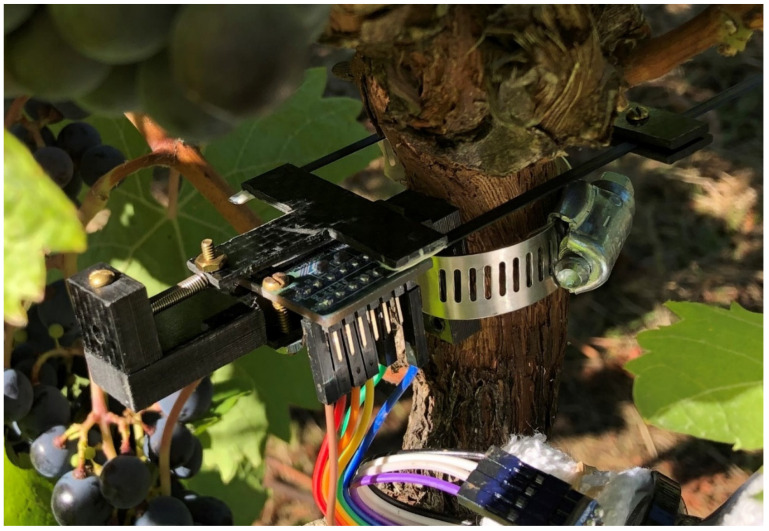
This figure shows a field-deployed dendrometer device monitoring the diameter of a grape vine to determine the amount of water being absorbed by the plant.

Dendrometers [58] (Figure 19) are a plant-based tool that has shown potential to improve irrigation management in high-value woody perennial crops (e.g., trees and vines). A dendrometer continuously measures small fluctuations in stem diameter; this has been directly correlated to water stress measurements using traditional methods. While plant-based measures of water deficits are the best measures of water stress, current dendrometer methods are imprecise due to mechanical hysteresis and thermal expansion. Loom manages the linear magnetic encoder used to track 0.5 micron fluctuations in stem diameter and supporting sensors including air temperature and humidity. It also manages all support functions like logging, LoRa telemetry to a 4G hub, and power savings (the diagram in Figure 12 is based on this project). The system was developed by an undergraduate engineering team, yielded an undergraduate first-author publication, utility patent [59], and is in use at Oregon State University, University of British Columbia, and Biosphere 2 to help researchers to better understand plant–irrigation relationships.

### 5.3. eGreenhouse, Robotically Positioned Sensor Package

**Figure 20:**
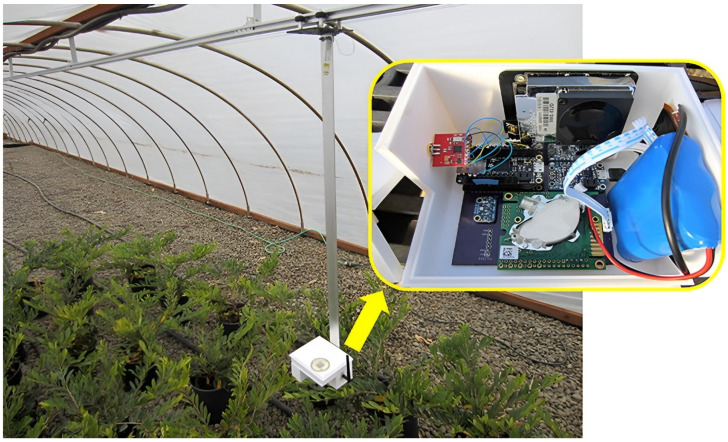
This figure shows an eGreenhouse sensor package mounted on the rail in a greenhouse with a closeup view inside the enclosure showing components.

The eGreenhouse sensor package (as seen in Figure 20) is designed to measure the spatial and temporal distribution of CO_2_ concentrations in greenhouses. These data are crucial for various fields including soil science, agriculture, and atmospheric science. Traditionally, CO_2_ detection has relied on stationary sensors with low spatial resolution. What if you could build up a profile of CO_2_ concentrations along a path? The system is comprised two Loom-configured devices: a data hub and liner rail motor controller, and a remote mobile sensor package. The hub controls a motor to transport the remote sensor package down a track. The hub also sends requests at user-specified intervals to the remote sensor package to report data. Relayed data are saved to an SD card and uploaded online for the sensor package, Loom-supported integration of a non-dispersive infrared (NDIR) CO_2_ sensor, combined with pre-existing temperature, relative humidity, and luminosity sensing onto a single logging device, providing high-resolution data of the greenhouse environment along the rail path. The resulting system was designed and published by a visiting postdoctoral student and two undergraduate engineering students in HardwareX [60]. Systems like these could provide significant spatiotemporal data for research, monitoring, and management for greenhouses. 

### 5.4. LilyPad

**Figure 21:**
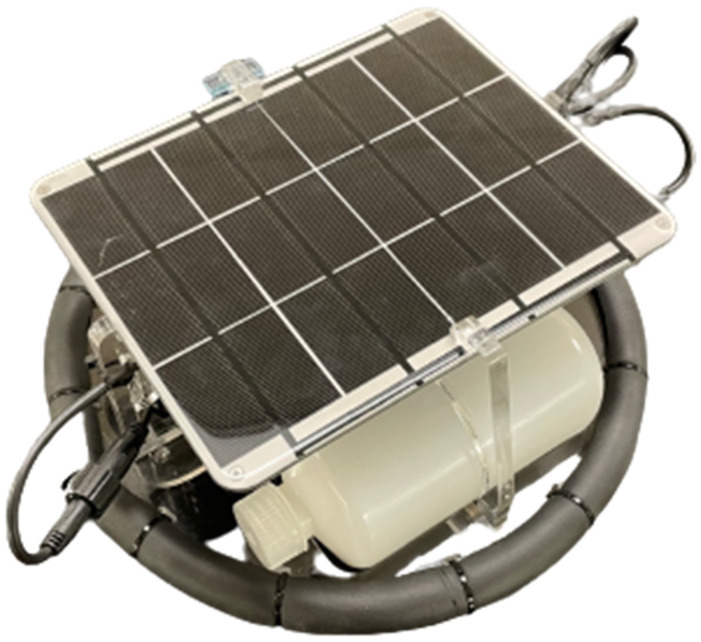
This figure shows a LilyPad prototype device.

Fresh water is among the most critical resources. The Lilypad [61] (as seen in Figure 21) aims to provide live data to assist in monitoring water loss and water management in freshwater reservoirs. The device collects water temperature, air temperature, air humidity, and solar energy per square meter, logging the data locally to an SD card and sending the data over 4G for live monitoring. It was designed by an undergraduate electrical engineering student and an undergraduate mechanical engineering student with intent for use in Africa. Most of the programming and electrical design aspects already existed in Loom. Only a new light sensor with application-specific requirements needed to be added. LilyPad is being used on freshwater reservoirs in Ontario, Canada to monitor the effects of solar panels deployed on the surface. The Lilypad aims to be a low-cost alternative to traditional methods of long-term water surface monitoring buoys.

### 5.5. Weed Warden

**Figure 22:**
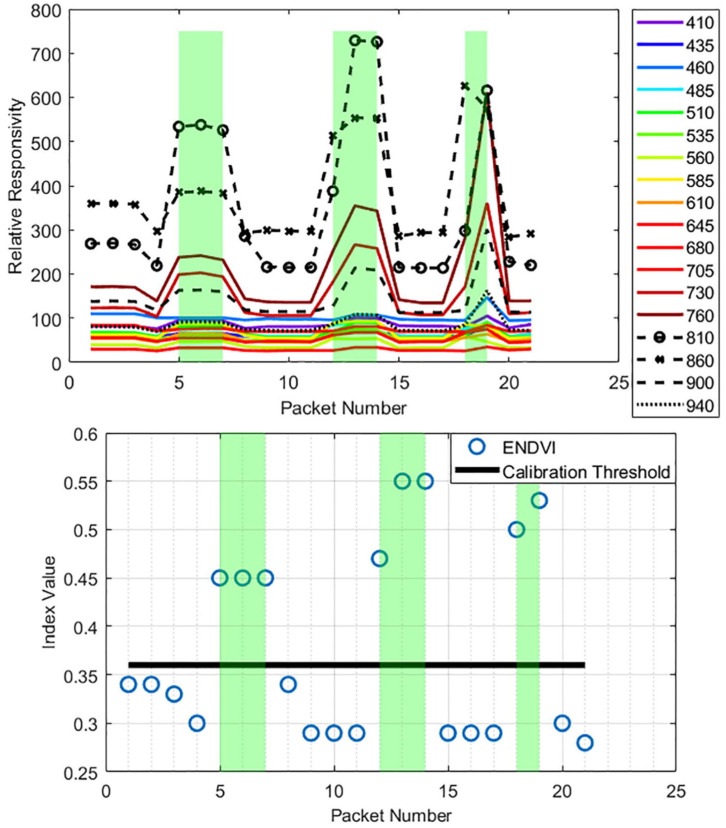
The top plot shows the Weed Warden sensor response when hovering over dirt versus plant sample (highlighted in green). The bottom plot shows the calculated index (blue) intersected with the set threshold (black) used to activate the 12 V relay to activate an intervention mechanism (e.g., spray nozzle, tillage arm, etc.).

Controlling the weed population in agriculture ensures water, nutrients, and other major resources are maximized. Current methods often resort to harmful herbicides, which are costly, pose health risks, and stimulate plant resistance to existing herbicides. This requires stronger chemical interventions each passing year. In fallow fields, it is desired for no plants to grow to give the soil time to recover. Strategic spot application herbicide or other interventions such as steam or tillage would significantly reduce the negative impacts of traditional management methods. Weed Warden [62] consists of a spectroscopy sensor, OPEnS Lab Hypnos Board, 5 V battery, and Adafruit Feather M0 with SD storage. A team of electrical engineering and biological and ecological engineering students integrated the SparkFun spectral triad sensor AS7265x [63] into Loom. The sensor is mounted to a mobile system to take readings of the ground and determine if weeds in fallow fields are present. If weeds are present, this will trigger a 12V relay on the Hypnos board that would power a spray nozzle to dispose of the weed (this functionality is demonstrated in Figure 13). The method used to determine if a weed is present is called the normalized difference vegetation index (NDVI). This algorithm compares the near-infrared (which vegetation strongly reflects) and red light (which vegetation absorbs) to output a value between −1 and +1. By setting a benchmark NDVI value on a patch of bare dirt and comparing that value to a new value, the presence of plants can be accurately determined; this is shown in Figure 22. This prototype does not yet distinguish between weeds and other plants. However, the objective to target all plants for fallow field management provided a more accessible proof of concept and significant real-world application. This effort yielded a peer-reviewed paper with three undergraduate co-authors.

## 6. Discussion

The purpose of this section is to compare our approach with other similar and established open-source libraries. Of the existing open-source sensor solutions, libraries such as the Adafruit Unified Sensor Driver [64] and ModularSensors are similar to Loom and attempt to facilitate more efficient usage of sensors. While the Adafruit Unified Sensor Driver exclusively supports sensors, the ModularSensors library also supports radio telemetry, internet logging platforms, and debugging tools. ModularSensors does have key advantages over Loom for many applications, most especially a wider variety of hydrological monitoring sensors, a wider variety of supported radios, and one additional internet publishing platform, Monitor my Watershed [65]. ModularSensors has been available since 2017 and has a significantly larger and more established userbase.

In contrast, Loom accommodates a broader scope of sensors across a variety of environments such as accelerometers, carbon dioxide (CO_2_) sensors, air quality (PM2.5 PM10), and multispectral optical sensors (Appendix A) with less comprehensive support for water quality sensing. Loom’s actuator support also poses a significant advantage for many applications requiring mechatronic response to environmental stimuli.

While ModularSensors supports debugging applications through a serial monitor, Loom’s SD card debugging framework uniquely allows for diagnosing problems on devices where a serial connection to a computer in the field is not feasible. Loom also allows users to find and fix instances where dynamically allocated memory is not cleared properly resulting in an over-usage of memory ending in a crash. Loom combats this through generating function usage summaries that log exactly how much memory each function is using.

Loom’s Manager framework streamlines and standardizes integration of new components like sensors and actuators by only including the module header and completing its constructor. The rest is handled by the Manager, eliminating the need to manage individual components.

Loom also has support for publishing data via MQTT to MongoDB and ThingSpeak [66]. ModularSensors does not support MongoDB logging but does support ThingSpeak. ThingSpeak supports a maximum of publishing to eight distinct data fields, while MongoDB supports packets up to two thousand bytes (limited arbitrarily by the Loom support code and adjustable). Loom’s MQTT implementation is agnostic of the target database. Instead of rewriting Loom’s MQTT Manager, only the server credentials to the target broker need to be modified.

Loom’s code structure is more succinct and streamlined. A comparison of the code required to obtain an Atlas Scientific EZO-CO_2_ sensor [67], supported in both Loom and ModularSensors, working with a standard cycle of recording sensor measurements, packaging the retrieved data into JSON and logging the data to an SD card is about half as many lines of code in Loom versus the ModularSensors equivalent.

Loom’s Hypnos class and the ModularSensors’s Logger class perform similar functions including real-time clock, low-power standby management, and SD logging. The Hypnos class also has functionality to physically cut power to some or all connected peripherals, allowing for even greater power savings.

Loom utilizes statically allocated c-style string buffers whereas ModularSensors utilizes Arduino’s built-in string implementation, which if used too frequently can result in memory fragmentation and inevitably a crash due to RAM depletion (which we encountered when developing Loom). Another distinguishing approach comparing these two libraries is that Loom pivoted away from using the WDT to recover from hard-faults and hanging states, a strategy often employed in these systems and used in ModularSensors.

Finally, Loom supports the ability to interact with many aspects of a sensor-actuator device over a Wi-Fi connection (either via router or local hotspot) utilizing the Max8 application. This functionality provides accessible data manipulation and control of devices that have been used in educational workshops for high school, undergraduate, scientific, and community programs.

## 7. Conclusions

The availability of more open-source options to rapidly prototype in situ sensor systems is crucial for supporting scientific observations in the face of changing climate patterns, hazards, and rising human population. While there are other open-source frameworks that address similar needs, Loom contains unique features (in addition to sensor data acquisition, logging, and telemetry) including actuation control, interactive data applications for education and scientific workshops, field and classroom utilities, diagnostic tools, and a streamlined integration workflow.

The motivation behind this abstraction structure is to enable a low floor to accessibility, while maintaining high overhead for customization and extensibility. While the extent to how much more accessible this approach is compared to others has not been formally studied, the variety and complexity of peer-reviewed systems being designed mostly by undergraduate researchers is considerable evidence for Loom’s usability.

One notable significant development bottleneck is the dependence, time, and cost to validate these systems on physical hardware. Using AI-based models such as Digital Twins [68] could accelerate our development process by simulating different hardware and environments.

Moving forward, Loom will continue to expand its support for various sensors and actuators. Strengths and weaknesses of Loom will be compared to preexisting datalogging solutions, commercial and open-source, to categorically evaluate the viability of Loom in various applications.

## 8. Patents

The specific sensor design of the dendrometer mentioned in this manuscript maintains a utility patent issued to Oregon State University on 8 June 2023, with the number OSU-21-38.

## Data Availability

The data presented in this study are openly available in Loom at https://doi.org/10.5281/zenodo.11318101 (accessed on 23 May 2024).

## Appendix A

**Table A1:** This table provides a list of I2C sensors currently supported by the Loom framework.

Sensor Name	Datasheet URL	Resolution	Range	Accuracy
ADS1115 16-Bit ADC	https://cdn-shop.adafruit.com/datasheets/ads1115.pdf (Accessed 23 May 2024)	16 bits	Depends on gain: See datasheet	N/A
Adafruit AS7262 6-Channel Visible Light/Color Sensor	https://ams.com/documents/20143/36005/AS7262_DS000486_5-00.pdf (Accessed 23 May 2024)	16 bits	450–650 nm	±5 nm
SparkFun AS7263 Spectral Sensor	https://cdn.sparkfun.com/assets/1/b/7/3/b/AS7263.pdf (Accessed 23 May 2024)	16 bits	610–860 nm	±5 nm
SparkFun AS7265x Spectral Triad Sensor	https://cdn.sparkfun.com/assets/c/2/9/0/a/AS7265x_Datasheet.pdf (Accessed 23 May 2024)	16 bits	410–940 nm	±10 nm
EZO-CO_2_™ Embedded Carbon Dioxide Sensor	https://files.atlas-scientific.com/EZO_CO2_Datasheet.pdf (Accessed 23 May 2024)	1 ppm	0–10,000 ppm	(±5%) + (±50 ppm)
EZO™ Dissolved Oxygen Circuit	https://files.atlas-scientific.com/DO_EZO_Datasheet.pdf (Accessed 23 May 2024)		0.00–100 mg/L	±0.05 mg/L
EZO™ ORP Circuit	https://files.atlas-scientific.com/ORP_EZO_Datasheet.pdf (Accessed 23 May 2024)	N/A	−1019.9 mV–1019.9 mV	±1 mV
EZO™ pH Circuit	https://files.atlas-scientific.com/pH_EZO_Datasheet.pdf (Accessed 23 May 2024)	0.001 pH	0.001–14.000 pH	±0.002 pH
EZO-RGB™ Embedded Color Sensor	https://files.atlas-scientific.com/EZO_RGB_Datasheet.pdf (Accessed 23 May 2024)	24 bits	0–65,535 LuX	N/A
K30 CO_2_ Sensor	https://rmtplusstoragesenseair.blob.core.windows.net/docs/publicerat/PSH0131.pdf (Accessed 23 May 2024)	N/A	0–5000 ppm	±30 ppm
MB1232 Ultrasonic Sensor	https://maxbotix.com/pages/i2cxl-maxsonar-ez-datasheet (Accessed 23 May 2024)	1 cm	0 cm–765 cm	N/A
Adafruit MMA8451 Triple-Axis Accelerometer	https://cdn-shop.adafruit.com/datasheets/MMA8451Q-1.pdf (Accessed 23 May 2024)	14 bits	2 g–8 g (depending on mode)	±2.64%
MPU-6050 Six-Axis (Gyro + Accelerometer)	https://invensense.tdk.com/wp-content/uploads/2015/02/MPU-6000-Datasheet1.pdf (Accessed 23 May 2024)	16 bits	2 g–16 g	±2 g, ±4 g, ±8 g, ±16 g
MS580302BA01-00 Pressure Sensor	https://www.te.com/usa-en/product-MS580302BA01-00.datasheet.pdf (Accessed 23 May 2024)	0.024 mbar	300 mbar to 1.1 bar	±2.5 mbar
SEN55 PM, RH/T, VOC, and NOx Sensor	https://cdn.sparkfun.com/assets/5/b/f/2/8/Sensirion_Datasheet_SEN5x.pdf (Accessed 23 May 2024)	Multiple: See datasheet	Multiple: See datasheet	Multiple: See datasheet
SHT31 Temperature and Humidity	https://cdn-shop.adafruit.com/product-files/2857/Sensirion_Humidity_SHT3x_Datasheet_digital-767294.pdf (Accessed 23 May 2024)	0.01%RH/0.015 °C	0 to 100%RH/−40 to 125 °C	±2%RH/±0.3 °C
STEMMA Soil Moisture	https://cdn-learn.adafruit.com/downloads/pdf/adafruit-stemma-soil-sensor-i2c-capacitive-moisture-sensor.pdf (Accessed 23 May 2024)	N/A	200–2000 (very dry–very wet)	N/A
TSL2591 High Dynamic Range Light Sensor	https://ams.com/documents/20143/9331680/TSL2591_DS000338_7-00.pdf (Accessed 23 May 2024)	N/A	188–88,000 uLux	N/A
ZX Gesture Sensor	https://cdn.sparkfun.com/assets/learn_tutorials/3/4/5/XYZ_Interactive_Technologies_-_ZX_SparkFun_Sensor_Datasheet.pdf (Accessed 23 May 2024)	N/A	N/A	N/A

Key sensor metrics have been provided; however, the link to each datasheet will have additional information.

**Table A2:** This table provides a list of all additional sensors supported by the Loom framework.

Sensor Name	Datasheet URL	Protocol	Resolution	Range	Accuracy
ADS1232 Analog-to-Digital Converter	https://www.ti.com/lit/ds/symlink/ads1232.pdf (Accessed 23 May 2024)	SPI	24 bits	±1.5 V	N/A
MAX31856 Precision Thermocouple	https://www.analog.com/media/en/technical-documentation/data-sheets/MAX31856.pdf (Accessed 23 May 2024)	SPI	19 bit/0.0078125 °C	Refer to Table 1 in datasheet linked on right	±0.15%
MAX31865 Digital Resistance Converter	https://www.analog.com/media/en/technical-documentation/data-sheets/MAX31865.pdf (Accessed 23 May 2024)	SPI	15 bit/0.03125 °C	−40 to 125 °C	±0.05%/±0.5 °C
Decagon GS3 Soil Moisture Sensor	https://library.metergroup.com/Manuals/20429_GS3_Web.pdf (Accessed 23 May 2024)	SDI12	See Section 3.1 in datasheet linked datasheet	0.0–1.0 m^3^/m^3^	±0.03 m^3^/m^3^
Teros 10 Soil Moisture Sensor	https://publications.metergroup.com/Manuals/20788_TEROS10_Manual_Web.pdf (Accessed 23 May 2024)	Analog	0.001 m^3^/m^3^	0.00–0.64 m^3^/m^3^	±0.03 m^3^/m^3^
Teros 11 Soil Moisture Sensor	https://publications.metergroup.com/Manuals/20587_TEROS11-12_Manual_Web.pdf (Accessed 23 May 2024)	SDI12	Multiple: see Section 3.1 in datasheet	Multiple: see Section 3.1 in datasheet	Multiple: see Section 3.1 in datasheet
Teros 12 Soil Moisture Sensor	https://publications.metergroup.com/Manuals/20587_TEROS11-12_Manual_Web.pdf (Accessed 23 May 2024)	SDI12	Multiple: see Section 3.1 in datasheet	Multiple: see Section 3.1 in datasheet	Multiple: see Section 3.1 in datasheet
SDS011 Laser Particulate Matter Sensor	https://cdn-reichelt.de/documents/datenblatt/X200/SDS011-DATASHEET.pdf (Accessed 23 May 2024)	UART	0.3 μg/m^3^	0.0–999.9 μg/m^3^	±15%

Key sensor metrics have been provided; however, the link to each datasheet will have additional information.

**Table A3:** This table provides a list of all actuators supported by the Loom framework.

Sensor Name	Part #	Datasheet URL	Description
Adafruit Neopixel	WS2812B or SKC6812	https://www.adafruit.com/category/168 (Accessed 23 May 2024)	Supports any Adafruit Neopixel-based Device
Non-latching Relay	3191	https://cdn-shop.adafruit.com/product-files/3191/G5LE-14-DC3-Omron-datasheet-10841140.pdf (Accessed 23 May 2024)	Supports any relay that is controlled by toggling a pin at high or low
Adafruit Servo Driver	2928	https://www.adafruit.com/product/2928 (Accessed 23 May 2024)	8 channel Adafruit Servo drivers that allow for generic PWM control of servos
Adafruit Stepper Motor Driver	1483	https://www.adafruit.com/product/1438 (Accessed 23 May 2024)	DC Motor/Stepper Motor Driver Board

**Table A4:** This table provides a list of all additional hardware supported by the Loom framework.

Sensor Name	Part #	Product URL	Description
SARA-R4 LTE Shield	14997	https://www.sparkfun.com/products/14997 (Accessed 23 May 2024)	SparkFun SARA-R4 LTE shield for cellular connections

**Table A5:** This table provides a list of all officially supported Loom projects.

Project Name	Project Page	Description
WeatherChimes	https://github.com/OPEnSLab-OSU/WeatherChimes (Accessed 23 May 2024)	In situ remote weather station
LilyPad	https://github.com/OPEnSLab-OSU/Lilypad (Accessed 23 May 2024)	Floating watershed monitoring station
FloDar	https://github.com/OPEnSLab-OSU/FloDar (Accessed 23 May 2024)	Remote datalogger measuring flow rate (L/min) of water passing through a pipe for prolonged periods of time
Smart Rock	https://github.com/OPEnSLab-OSU/SmartRock (Accessed 23 May 2024)	Low-cost device to monitor remote streams
Evaporometer	https://github.com/OPEnSLab-OSU/Evaporometer (Accessed 23 May 2024)	Low-cost load cell-based rain gauge system
Dendrometer	https://github.com/OPEnSLab-OSU/Dendrometer (Accessed 23 May 2024)	Effectively measure and log diurnal fluctuations in grape vines
eGreenhouse	https://github.com/OPEnSLab-OSU/eGreenHouse (Accessed 23 May 2024)	Lightweight and low-cost greenhouse sensor package
WeedWarden	https://github.com/OPEnSLab-OSU/WeedWarden (Accessed 23 May 2024)	Low-cost plant detection sensor that can be mounted on rovers or tractors.

**Table A6:** This table provides a list of uncommon words and abbreviations with their descriptions or full terms in the order they appear.

Abbreviation/Word	Full Term	Description
STEM	Science, technology, engineering, and mathematics	Common term used in educational literature and programs
LTE	Long-Term Evolution	Current generation of cellular data transmission
JSON	JavaScript Object Notation	Used to serialize data to a dictionary
SAMD21/51	N/A	Microcontroller from the SAM D21/DA1 family developed by Microchip
SAMD	N/A	Family of ARM^®^ processors developed by Microchip
LoRa	Long-Range, Low-Power Wide Area Network	Used for low-power radio transmission
SD	Secure Digital	Used for low-power radio transmission
Adalogger	N/A	Feather M0 formfactor board with built-in SD card reader/writer
ESP32	N/A	Microcontroller developed by Espressif systems
RAM	Random access memory	N/A
kB	Kilobytes	N/A
ATmega32u4	N/A	AVR^®^ microcontroller developed by Microchip
TI MSP430	N/A	General purpose microcontroller developed by Texas Instruments
IDE	Interactive Development Environment	N/A
PCB	Printed Circuit Board	N/A
I2C	Inter-Integrated Circuit	Used to communicate between the microcontroller and other peripherals
UART	Universal Asynchronous Receiver/Transmitter	Used to communicate between the microcontroller and other peripherals
SDI12	Serial Digital Interface at 1200 baud	Used to communicate between the microcontroller and other peripherals
SPI	Serial Peripheral Interface	Used to communicate between the microcontroller and other peripherals
PWM	Pulse Width Modulation	Used to control analog circuits with digital outputs
DC	Direct Current	N/A
LED	Light-Emitting Diode	N/A
SARA-R4	N/A	LTE module developed by u-blox
SIM	Subscriber Identity Module	Used to identify cellular connections with the cell network
RockBLOCK Mk2	N/A	Iridium satellite communication module
RFM	Radio Frequency Module	N/A
kbps	Kilobits per second	N/A
RTC	Real-time clock	Keeps track of the current time
TCA9548	N/A	I2C multiplexer developed by Texas Instruments
MPU6050	N/A	3-axis gyroscope and accelerometer
RGB	Red, green, blue	N/A
BEE222	Biological and Ecological Engineering 222	The course number and department that the shield was made for
DS3231	N/A	Real-time clock hardware developed by Analog Devices
CSV	Comma-separated values	File format used to store data
MQTT	Message Queuing Telemetry Transport	Used to upload data from a device to a remote server
Serial Monitor	N/A	Interface used to communicate between the device and a connected computer
UI	User interface	N/A
WDT	Watchdog Timer	Will reset the device if triggered to recover from a hung state
CO_2_	Carbon dioxide	N/A
NDIR	Non-dispersive infrared	N/A
NDVI	Normalized difference vegetation index	N/A
MongoDB	N/A	No-SQL database used to store device measurements
EZO™	N/A	Line of Atlas Scientific water sensors
C-style string	N/A	An array of characters in memory that terminated with null (0) byte

## References

[1] Communications-Wireless, Remote, Hard-Wired, Direct, or Two-Way Communication. http://www.campbellsci.com/communications.

[2] Messer H., Zinevich A., Alpert P. (2012). Environmental sensor networks using existing wireless communication systems for rainfall and wind velocity measurements. IEEE Instrum. Meas. Mag..

[3] Andres L., Boateng K., Borja-Vega C., Thomas E. (2018). A Review of In-Situ and Remote Sensing Technologies to Monitor Water and Sanitation Interventions. Water.

[4] Arnold C., Harms M., Goschnick J. (2002). Air Quality Monitoring and Fire Detection With The Karlsruhe Electronic Micronose KAMINA. Sens. J. IEEE.

[5] Grimaldi D., Marinov M. (2001). Distributed measurement systems. Measurement.

[6] Lee D.-D., Lee D.-S. (2001). Environmental gas sensors. IEEE Sens. J..

[7] Kim Y., Evans R.G., Iversen W.M. (2008). Remote Sensing and Control of an Irrigation System Using a Distributed Wireless Sensor Network. IEEE Trans. Instrum. Meas..

[8] Lee H.C., Banerjee A., Fang Y.-M., Lee B.-J., King C.-T. (2010). Design of a Multifunctional Wireless Sensor for In-Situ Monitoring of Debris Flows. IEEE T Instrum. Meas..

[9] Lempert R.J., Busch L., Brown R., Patton A., Turner S., Schmidt J., Young T. (2023). Community-Level, Participatory Co-Design for Landslide Warning with Implications for Climate Services. Sustainability.

[10] Pörtner H.-O., Roberts D.C., Poloczanska E.S., Intergovernmental Panel on Climate Change (2022). Summary for Policymakers. Climate Change 2022: Impacts, Adaptation, and Vulnerability. Contribution of Working Group II to the Sixth Assessment Report of the Intergovernmental Panel on Climate Change.

[11] Moser S.C. (2016). Can science on transformation transform science? Lessons from co-design. Curr. Opin. Environ. Sustain..

[12] Reed M.S. (2008). Stakeholder participation for environmental management: A literature review. Biol. Conserv..

[13] Blomkamp E., Howlett M., Mukherjee I. (2018). The Promise of Co-Design for Public Policy. Routledge Handbook of Policy Design.

[14] Aufdenkampe A.K., Damiano S.G., Hicks S., Horsburgh J.S. (2017). EnviroDIY ModularSensors: A Library to give Environmental Sensors a Common Interface of Functions for use with Arduino-Compatible Dataloggers. AGU Fall Meeting Abstracts.

[15] Ali A.S., Zanzinger Z., Debose D., Stephens B. (2016). Open Source Building Science Sensors (OSBSS): A low-cost Arduino-based platform for long-term indoor environmental data collection. Build. Environ..

[16] CR1000X: Measurement and Control Datalogger. https://www.campbellsci.com/cr1000x.

[17] Clement S., Spellman K., Oxtoby L., Kealy K., Bodony K., Sparrow E., Arp C. (2023). Redistributing Power in Community and Citizen Science: Effects on Youth Science Self-Efficacy and Interest. Sustainability.

[18] CRBasic Help-CRBasic Editor. https://help.campbellsci.com/crbasic/cr6/.

[19] What Is Arduino?. https://www.arduino.cc/en/Guide/Introduction.

[20] Seeed Studio Bazaar, The IoT Hardware Enabler. https://www.seeedstudio.com/.

[21] DFRobot Open-Source Hardware Electronics and Kits. https://www.dfrobot.com/.

[22] JSON. https://www.json.org/json-en.html.

[23] A. Industries, Adafruit Feather M0 Basic Proto-ATSAMD21 Cortex M0. https://www.adafruit.com/product/2772.

[24] (2020). Microchip, SAM D21/DA1, DS40001882F. https://ww1.microchip.com/downloads/en/DeviceDoc/SAM_D21_DA1_Family_DataSheet_DS40001882F.pdf.

[25] A. Industries, Adafruit Feather M0 with RFM95 LoRa Radio-900MHz. https://www.adafruit.com/product/3178.

[26] Adafruit Feather M0 WiFi-ATSAMD21 + ATWINC1500: ID 3010: $39.95: Adafruit Industries, Unique & fun DIY Electronics and Kits. https://www.adafruit.com/product/3010.

[27] A. Industries. Adafruit Feather M0 Bluefruit LE. https://www.adafruit.com/product/2995.

[28] A. Industries. Adafruit Feather M0 Adalogger. https://www.adafruit.com/product/2796.

[29] Adafruit Learning System Feather Specifications. https://learn.adafruit.com/adafruit-feather/feather-specification.

[30] Adafruit Learning System Relay Wings. https://learn.adafruit.com/adafruit-feather/relay-wings.

[31] Adafruit Learning System Adafruit 8-Channel PWM or Servo FeatherWing. https://learn.adafruit.com/adafruit-8-channel-pwm-or-servo-featherwing/overview.

[32] Adafruit Learning System Adafruit Stepper + DC Motor FeatherWing. https://learn.adafruit.com/adafruit-stepper-dc-motor-featherwing/overview.

[33] NeoPixels Products Category on Adafruit Industries. https://www.adafruit.com/category/168.

[34] A. Industries. Adafruit Ethernet FeatherWing. https://www.adafruit.com/product/3201.

[35] SparkFun LTE CAT M1/NB-IoT Shield-SARA-R4-CEL-14997-SparkFun Electronics. https://www.sparkfun.com/products/14997.

[36] RockBLOCK Mk2-Iridium SatComm Module-WRL-13745-SparkFun Electronics. https://www.sparkfun.com/products/13745.

[37] Ebrahimi K.K., Lunn G.R., Hudson B.M., Udell C., Selker J.S. Slide Sentinel: A Fully Automated, Low-Cost Landslide Monitoring System Using Real Time Kinematics. Proceedings of the AGU Fall Meeting 2019, AGU.

[38] Kampianakis E., Kimionis J., Tountas K., Konstantopoulos C., Koutroulis E., Bletsas A. (2014). Wireless Environmental Sensor Networking With Analog Scatter Radio and Timer Principles. IEEE Sens. J..

[39] SX1276/77/78/79—137 MHz to 1020 MHz Low Power Long Range Transceiver. https://cdn-shop.adafruit.com/product-files/3179/sx1276_77_78_79.pdf.

[40] Ameloot T., Van Torre P., Rogier H. (2018). A Compact Low-Power LoRa IoT Sensor Node with Extended Dynamic Range for Channel Measurements. Sensors.

[41] Augustin A., Yi J., Clausen T., Townsley W.M. (2016). A Study of LoRa: Long Range & Low Power Networks for the Internet of Things. Sensors.

[42] Chu M., Patton A., Roering J., Siebert C., Selker J., Walter C., Udell C. (2021). SitkaNet: A low-cost, distributed sensor network for landslide monitoring and study. HardwareX.

[43] Z9-T, Digi-Key Electronics. https://www.digikey.com/en/products/detail/freewave-technologies/Z9-T/9963074.

[44] Embeddable Industrial-Grade Radio Module. https://www.freewave.com/wp-content/uploads/2019/07/FreeWave-LDS0006AA-ZumLink-Z9-C-T-900-Series-Serial-Radio-Module-DS-Jul-2019.pdf.

[45] 2.4 GHz Transceiver IC-nRF24L01+-COM-00690-SparkFun Electronics. https://www.sparkfun.com/products/690.

[46] In-Depth: How nRF24L01 Wireless Module Works & Interface with Arduino. Last Minute Engineers. https://lastminuteengineers.com/nrf24l01-arduino-wireless-communication/.

[47] Nguyen B., Goto B., Selker J.S., Udell C. (2021). Hypnos board: A low-cost all-in-one solution for environment sensor power management, data storage, and task scheduling. HardwareX.

[48] Milford C., Udell C., Selker J.S. Smart Rock: Low Cost, User Friendly Stream Monitoring. Proceedings of the the Fall Meeting 2022, AGU.

[49] Kang K., Kerr A.C., Smith M., Brady C.G., Koontz N., Selker J.S., Udell C. Loom, A Simple Modular Framework for Rapid Prototyping Environmental Sensors, Actuators, and Data Collection. Proceedings of the AGU Fall Meeting 2020, AGU.

[50] “What Is Polymorphism?|Definition from TechTarget,” WhatIs. https://www.techtarget.com/whatis/definition/polymorphism.

[51] Greiman B. greiman/SdFat. https://github.com/greiman/SdFat.

[52] RadioHead: RadioHead Packet Radio Library for Embedded Microprocessors. https://www.airspayce.com/mikem/arduino/RadioHead/.

[53] MessagePack: It’s Like JSON. But Fast and Small. https://msgpack.org/.

[54] Eclipse Mosquitto. https://mosquitto.org/.

[55] Node-RED. https://nodered.org/.

[56] What Is Max?|Cycling ’74. https://cycling74.com/products/max.

[57] Woo W., Richards W., Selker J., Udell C. (2023). WeatherChimes: An Open IoT Weather Station and Data Sonification System. HardwareX.

[58] Clonch C., Huynh M., Goto B., Levin A., Selker J., Udell C. (2021). High precision zero-friction magnetic dendrometer. HardwareX.

[59] Clonch C., Goto B., Huynh M., Selker J., Udell C. (2023). Magnetic Dendrometer Apparatus and Corresponding Method. https://patents.google.com/patent/US20230175830A1/en?q=(Dendrometer)&before=publication:20230608&after=publication:20230608.

[60] Levintal E., Kang K.L., Larson L., Winkelman E., Nackley L., Weisbrod N., Selker J.S., Udell C.J. (2021). eGreenhouse: Robotically positioned, low-cost, open-source CO_2_ analyzer and sensor device for greenhouse applications. HardwareX.

[61] Home·OPEnSLab-OSU/Lilypad Wiki. https://github.com/OPEnSLab-OSU/Lilypad/wiki.

[62] Duncan L., Miller B., Shaw C., Graebner R., Moretti M.L., Walter C., Selker J., Udell C. (2022). Weed Warden: A low-cost weed detection device implemented with spectral triad sensor for agricultural applications. HardwareX.

[63] Smart 18-Channel VIS to NIR Spectral_ID 3-Sensor Chipset with Electronic Shutter. https://cdn.sparkfun.com/assets/c/2/9/0/a/AS7265x_Datasheet.pdf.

[64] Adafruit Unified Sensor Driver. Adafruit Industries, 18 November 2023. https://github.com/adafruit/Adafruit_Sensor.

[65] Monitor My Watershed. WikiWatershed. https://wikiwatershed.org/monitor/.

[66] IoT Analytics-ThingSpeak Internet of Things. https://thingspeak.com/.

[67] EZO-CO2^TM^ Embedded Carbon Dioxide Sensor. Atlas Scientific. https://atlas-scientific.com/probes/co2-sensor/.

[68] Kutscher V., Martins T.W., Olbort J., Anderl R. (2021). Concept for Interaction of Hardware Simulation and Embedded Software in a Digital Twin Based Test Environment. Procedia CIRP.

